# Immune Synapse Residency of Orai1 Alters Ca^2+^ Response of T Cells

**DOI:** 10.3390/ijms222111514

**Published:** 2021-10-26

**Authors:** Orsolya Voros, György Panyi, Péter Hajdu

**Affiliations:** 1 Department of Biophysics and Cell Biology, Faculty of Medicine, University of Debrecen, Debrecen, Egyetem tér 1, 4032 Debrecen, Hungary; voros.orsolya@med.unideb.hu (O.V.); panyi@med.unideb.hu (G.P.); 2 Department of Biophysics and Cell Biology, Faculty of Dentistry, University of Debrecen, Nagyerdei krt. 98, 4032 Debrecen, Hungary

**Keywords:** Orai1, immunological synapse, calcium signaling, STIM1, SAP97

## Abstract

CRAC, which plays important role in Ca^2+^-dependent T-lymphocyte activation, is composed of the ER-resident STIM1 and the plasma membrane Orai1 pore-forming subunit. Both accumulate at the immunological synapse (IS) between a T cell and an antigen-presenting cell (APC). We hypothesized that adapter/interacting proteins regulate Orai1 residence in the IS. We could show that mGFP-tagged Orai1-Full channels expressed in Jurkat cells had a biphasic IS-accumulation kinetics peaked at 15 min. To understand the background of Orai1 IS-redistribution we knocked down STIM1 and SAP97 (adaptor protein with a short IS-residency (15 min) and ability to bind Orai1 N-terminus): the mGFP-Orai1-Full channels kept on accumulating in the IS up to the 60th minute in the STIM1- and SAP97-lacking Jurkat cells. Deletion of Orai1 N terminus (mGFP-Orai1-Δ72) resulted in the same time course as described for STIM1/SAP97 knock-down cells. Ca^2+^-imaging of IS-engaged T-cells revealed that of Orai1 residency modifies the Ca^2+^-response: cells expressing mGFP-Orai1-Δ72 construct or mGFP-Orai1-Full in SAP-97 knock-down cells showed higher number of Ca^2+^-oscillation up to the 90th minute after IS formation. Overall, these data suggest that SAP97 may contribute to the short-lived IS-residency of Orai1 and binding of STIM1 to Orai1 N-terminus is necessary for SAP97-Orai1 interaction.

## 1. Introduction

In the adaptive immune response, the physical interaction between a T cell and an antigen presenting cell (APC) leads to the polarization of the T cell, with accumulation of cell surface proteins and signaling molecules at the T cell–APC contact site, forming a highly organized signaling platform known as the immunological synapse (IS) [1,2,3]. The encounter with APCs, where a specific antigen binding to the TCR/CD3 complex initiates different signaling cascades, results in T cell activation [4,5]. In this signaling event, the elevated Ca^2+^ concentration is due to the IP_3_ binding to the IP_3_ receptors in the endoplasmic reticulum (ER) and the releasing the Ca^2+^, that is followed by the opening of CRAC (Ca^2+^ release activated Ca^2+^ channels) channels composed of the pore-forming subunit Orai1 and the ER-resident activator STIM1 [6,7,8]. The calcium influx requires a concerted interplay between CRAC channels and depolarization-activated Kv1.3 and Ca^2+^-activated KCa3.1 potassium channels [9,10,11]. The former provides the influx pathway for calcium and the latter maintain a negative membrane potential, which generates the driving force for calcium entry and results in T cell proliferation. It has been demonstrated that expression of these ion channels, such as Kv1.3, KCa3.1 and Orai1 along with STIM1 is increased in activated human T cells, and they are involved in the Ca^2+^ signaling of T cells upon activation [10,12,13,14,15].

As mentioned above, the key step of T cell activation is the influx of Ca^2+^ ions through the CRAC channels. Depletion of the ER as a Ca^2+^-store is sensed by STIM1 subunit, which is localized in the ER membrane. STIM1 undergoes conformational changes, multimerize and translocate to the plasma membrane (PM), where they activate the PM-resident, pore-forming domain, Orai1 [16,17,18,19]. The channel opening is mediated by the interaction of the CRAC activation domain (CAD) on STIM1 with binding domains on the N- (73–91 aa) and C-termini (342–448 aa) of Orai1. It has been demonstrated that W76 (tryptophane) together with residues 73–75 of Orai1serve as a hotspot within the binding interface for interaction with STIM1 [6,20,21].

It has been reported that the Orai1 and STIM1 accumulates in the IS [14,22,23]. It was also shown that the Orai1 redistributes to the synapse when non-conducting mutant of Orai1 (E106) was expressed both in Jurkat and human primary T cells [14]. The calcium influx through CRAC channels organizes the actin filaments and facilitates retrograde actin flow in the IS, and therefore leads to ER redistribution and diffusion of Orai1 and STIM1 in the IS. The changes in actin organization and dynamics are a consequence of Ca^2+^ flux inhibition through CRAC channels, not the removal of Ca^2+^ itself [24]. However, the exact mechanism of Orai1 redistribution to the IS is still unknown.

It was reported that the Orai1 has two isoforms: the longer Orai1α consist of 301 amino acids (aa) and the shorter Orai1β misses the first 70 aa [25]. The Orai1α channel regarded as the wild-type variant is composed of six subunits [26,27,28]. Each subunit contains four alpha-helical transmembrane domain (TM) with intracellular N- and C-terminals and selectivity filter at position 106 [19]. Mutations in Orai1 cause defects in the immunity by abrogating CRAC channel activity. It was demonstrated that modified CRAC channel function leads to immunodeficiency, infections, T cell activation defects, congenital and global respiratory muscle insufficiency [29]. Therefore, studies on different phenotypes of Orai1 are very important to reveal the CRAC-related channelopathies.

In this study, we investigated the kinetics of Orai1 accumulation in the immunological synapse. We showed that Orai1 redistributed to the synapse and had a maximum accumulation at 15 min after IS formation. The N-terminal truncation of Orai1 (which reflecting situation of Orai1β isoform) or the knock-down of STIM1 led to retained accumulation dynamics of Orai1 subunits up to the 60th minute. The downregulation of SAP97 expression also led to a gradual increase in the frequency of Orai1 polarized cells up to the 60th minute. Ca^2+^-signaling of IS engaged cells was vastly different: cells with sustained Orai1 IS-accumulation (Jurkat cells expressing N-terminal mutant Orai1 or Orai1-Full in SAP97 knocked-down Jurkat cells) displayed oscillatory Ca^2+^-response up to the 90th minute after IS formation unlike control cells. These results suggest that Orai1 short-lived residence shape the Ca^2+^-response of T cells, and needed for appropriate physiological activation of lymphocytes.

## 2. Results

### 2.1. Kinetics of Orai1 Redistribution into the Immunological Synapse

**Figure 1:**
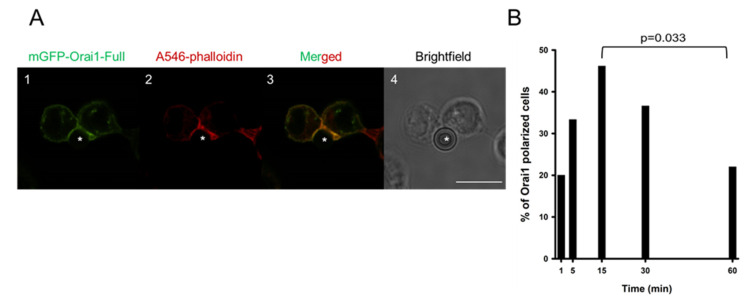
Redistribution of mGFP-Orai1-Full into the IS. Representative confocal images of the immune synapse formation between Jurkat cells expressing mGFP-tagged Orai1-Full channel and CD3-CD28 antibody coated beads. (**A1**) mGFP-tagged Orai1-Full channels are shown in green. (**A2**) Alexa-546 phalloidin stain of Jurkat cell (red) is shown (an IS formation indicator). (**A3**) Merged image of Orai1 (green) and Alexa-546 phalloidin (red) fluorescence signals. (**A4**) Brightfield image of mGFP-Orai1-Full channel expressing cell in Aa-Ac. The bead is indicated with an asterisk. Scale bar is 10 µm. (**B**) The percentage of cells showing polarized Orai1-Full expression upon the IS formation with CD3-CD28 antibody coated beads at 1st, 5th, 15th, 30th and 60th minute. 30 cells were analyzed at each time point.

Residency time of different ion channels (Kv1.3, KCa3.1 and Orai1) of T cells in the IS is presumed to be essential for shaping the Ca^2+^-dependent activation [30]. Previously it was described that Orai1 channels along with the STIM1 redistributed to the IS between a T cell and an APC [14]. To study the kinetics of Orai1 channel accumulation in the IS, we established Orai1 stably expressing cell line in Jurkat E6-1 cells. First, we tagged Orai1 channel with mGFP, and using retroviral transduction (for details see Materials and Methods) mGFP-Orai1-Full construct was expressed in Jurkat cells. IS-formation was initiated between anti-CD3 and anti-CD28 antibody coated beads and mGFP-Orai1-Full expressing cells, and the percentage of Orai1-polarized cells was determined at five subsequent time points (1, 5, 15, 30 and 60 min) (Figure 1A). To confirm the development of IS formation F-actin polymerization was monitored with the help of Alexa-546 phalloidin staining, and the accumulation ratio (AR) was introduced to decide if a T cell exhibited Orai1-polarization. To reduce false-positive detection rate, we set the threshold value for AR to 1.5, i.e., cells having AR value lower than 1.5 were defined as non-polarized (see Szilagyi et al. [13]). In the analysis, those cells were included which had F-actin polarization (AR value calculated for actin/phalloidin was higher than 1.5). For each time point we analyzed at least 30 cells (recorded at least on three consecutive days), and we determined the percent of cells with Orai1-accumulation in the IS (Figure 1B). 1 min after cell-bead encounter formation 20% of T cells showed Orai1-accumulation in the IS, and the portion of Orai1-polarized cells peaked at the 15th minute. At later time points (30 and 60 min) the percent of cells exhibiting Orai1 IS-redistribution declined gradually (Figure 1B). Next we studied how Orai1 is steered to the IS in Jurkat T cells.

### 2.2. STIM1 Controls the IS-Dwelling Time of Orai1

**Figure 2:**
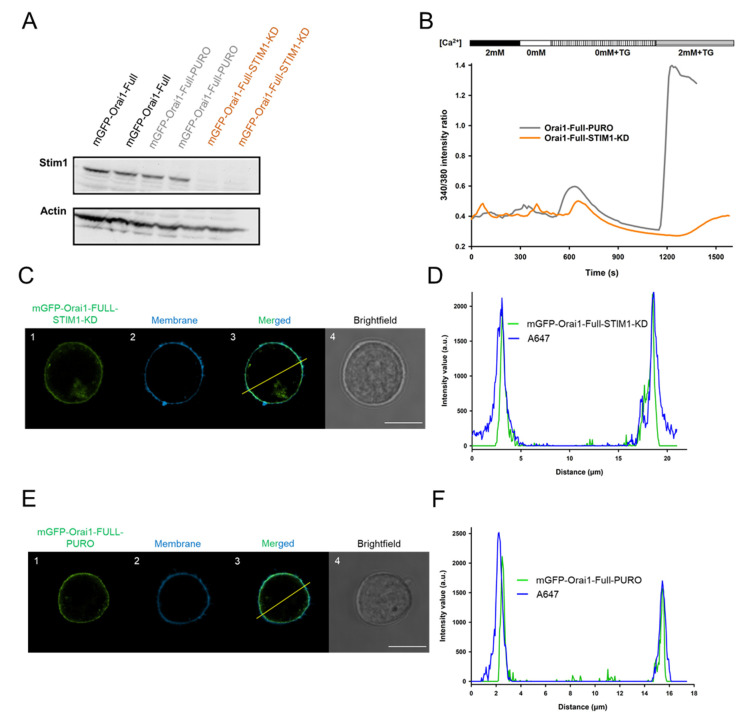
Knockdown of STIM1 in Jurkat cells expressing mGFP-Orai1-Full resulted in nonfunctional channels. (**A**) Western blot experiments of mGFP-Orai1-Full expressing Jurkat (black), mGFP-Orai1-Full-PURO (*grey*) and mGFP-Orai1-Full-STIM1-KD (orange) cells probed with a specific antibody against STIM1 (the expected size 85–88 kDa, each sample was loaded in duplicates). Actin antibody was used as an expression control with an approximate size of 42 kDa. (Knock-down efficiency was ca. 95%.) (**B**) Cytosolic Ca^2+^ measurements using FURA-2 in mGFP-Orai1-Full-PURO cells stably expressing mGFP-Orai1-Full channels (grey) and in Jurkat cells missing the Orai1 activator STIM1 protein (mGFP-Orai1-Full-STIM1-KD) stably expressing mGFP-Orai1-Full channels (orange). Cytosolic Ca^2+^ measurement based on the 340 nm/380 nm intensity ratio of FURA-2. The representative traces are the average of 18 cells (mGFP-Orai1-Full-PURO) and 8 cells (mGFP-Orai1-Full-STIM1-KD). (**C**) Typical confocal images of mGFP-Orai1-Full-STIM1-KD cells. (**C1**,**E1**) mGFP signal of Orai1-Full channel in STIM1-KD or PURO cells (*green*), (**C2**,**E2**) The Alexa Fluor™ 647 NHS Ester fluorescence of the plasma membrane (blue) in mGFP-Orai1-Full-STIM1-KD and mGFP-Orai1-Full-PURO cells. (**C3**,**E3**) The merge of green and blue channels in mGFP-Orai1-Full-STIM1-KD and mGFP-Orai1-Full-PURO cells, please note the overlapping of them. (**C4**,**E4**) Brightfield image of the mGFP-Orai1-Full channel expressing STIM1-KD or PURO cells in Ca-Cc or Ea-Ec. Scale bar is 10 µm. (**D**,**F**) The pixel-by-pixel intensity profile analysis of the mGFP-Orai1-Full-STIM1-KD and mGFP-Orai1-Full-PURO cell along the line indicated in the merged image (**C3**,**E3**): green: mGFP-Orai1, blue: membrane, Alexa-647 NHS ester.

The necessitous step of Orai1 activation and function is its coupling to the STIM1 subunit localized in the ER. Previously, it was reported that STIM1 is accumulated in the IS that is inevitable for adequate Ca^2+^ signaling [18,31]. Furthermore, STIM1 was also reported to guide the trafficking of Orai1 subunit to the plasma membrane: both suboptimal and supraoptimal expression of STIM1 led to low membrane targeting of Orai1 [32,33]. To understand the function of STIM1-Orai1 interaction in Orai1 IS-redistribution, we established an mGFP-Orai1-Full cell line, in which we knocked down the STIM1 gene (mGFP-Orai1-Full-STIM1-KD). As a control for transduction the pLKO.1-puro plasmid was applied, which contains shRNA sequence that does not target any known genes (mGFP-Orai1-Full-PURO). To test the efficiency of STIM1 knock down, the STIM1 expression level was assessed using western blotting. Cell lines transduced with STIM1 specific shRNA encapsulating virions the intensity of STIM1 band was far lower as compared to transduction control (mGFP-Orai1-Full-PURO) and mGFP-Orai1-Full cells (Figure 2A). On the other hand, we assessed the membrane targeting of mGFP-Orai1-Full subunits: the membrane was labelled with Alexa-647 succinimidyl ester (non-specifically) and the line intensity profile was evaluated for mGFP-Orai1-Full-PURO and mGFP-Orai1-Full-STIM1-KD cell lines (Figure 2C,E). Figure 2D,F noticeably indicate the membrane localization of Orai1 subunit: the fluorescent signal of membrane staining and mGFP-Orai1-Full proteins are overlapping.

For functional checkup, Ca^2+^ signaling of mGFP-Orai1-Full-PURO and mGFP-Orai1-Full-STIM1-KD Jurkat cells were characterized by the measurements of the cytosolic Ca^2+^ changes using FURA-2 ratiometric imaging. A typical time-course of the Ca^2+^ measurement is shown in Figure 2B: first, the cells were bathed in 2 mM Ca^2+^ solution then the extracellular solution was changed for absolute 0 mM Ca^2+^ containing solution to test the integrity of the plasma membrane and viability of cells. Then thapsigargin (TG, 1 µM) in Ca^2+^-free solution was added to cells to empty intracellular Ca^2+^ store, which triggered the assembly of STIM1 and Orai1. Upon re-addition of 2 mM Ca^2+^ in the presence of TG the Ca^2+^ current through the pore formed by Orai1 and activated by STIM1 could be detected. The control cells (mGFP-Orai1-Full-PURO) had a typical Ca^2+^ response seen for mGFP-Orai1-Full (please see also Figure 4B) and—as expected and published before [14,34,35]—STIM1 knock-down completely abolished Ca^2+^-response of cells through CRAC channels (Figure 2B).

**Figure 3:**
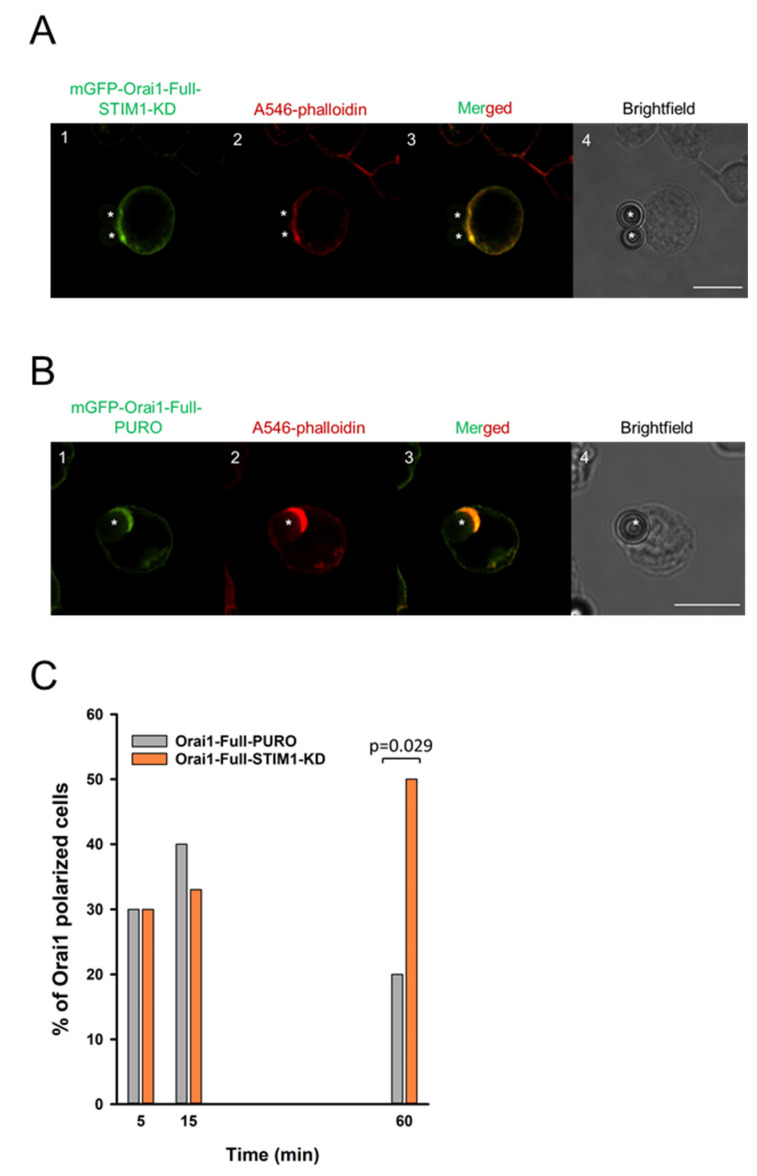
The Orai1-Full channel in STIM1 knockdown cells show unrelenting accumulation into the immunological synapse. Confocal images of the immune synapse formed between Jurkat cells expressing the mGFP-Orai1-Full channels (**A**) in STIM1-KD and (**B**) PURO cells and CD3-CD28 antibody coated beads. (**A1**,**B1**) The mGFP-tagged Orai1-Full channels in STIM1-KD and PURO cells are shown in green. (**A2**,**B2**) Alexa-546 phalloidin (red) label of cells (IS reporter). (**A3**,**B3**) Merged images of green (Orai1) and red (phalloidin) fluorescence signals. (**A4**,**B4**) Brightfield images of STIM1-KD (**A1**–**A3**) and PURO (**B1**–**B3**) cells expressing the mGFP-Orai1-Full channels. Beads are indicated with an asterisk. Scale bar is 10 µm. (**C**) The percentage of cells with the polarized Orai1-Full channel distribution upon IS formation with CD3-CD28 antibody coated beads at 5th, 15th and 60th minute in mGFP-Orai1-Full-STIM1-KD (orange) and mGFP-Orai1-Full-PURO (grey) cells. 30 cells were evaluated at each time point.

Afterwards, time-dependence of IS-accumulation for Orai1 channels in mGFP-Ora1-Full-PURO and mGFP-Ora1-Full-STIM1-KD cell lines was determined. As detailed above, we generated IS between these two cell lines and CD3/CD28 antibody coated beads at three time points (5th, 15th and 60th minute) as before (shown in Figure 3A,B) and evaluated the fraction of cells displaying Orai1 IS-redistribution (and showing F-actin polarization at the bead-cell contact area). The time course of the Orai1 IS-accumulation for mGFP-Orai1-Full-PURO cells (Figure 3C) was similar to mGFP-Orai1-Full construct expressing cells (Figure 1B): percentage of Orai1-polarized cells peaked at 15 min (app. 40%) and dropped to ca. 25 percent just as seen for WT-Orai1. However, STIM1 knockdown modified the kinetics of Orai1 IS redistribution: the fraction of cells showing Orai1 accumulation in the IS increased up to 60th minute in mGFP-Orai1-Full-STIM1-KD cells (Figure 3C, *p* = 0.029). AR values for these mGFP-Orai1-Full-PURO and mGFP-Orai1-Full-STIM1-KD were also statistically different (*p* = 0.015, Figure S6).

### 2.3. N-Terminal Truncation of Orai1 Mimics STIM1 Knock-Down

**Figure 4:**
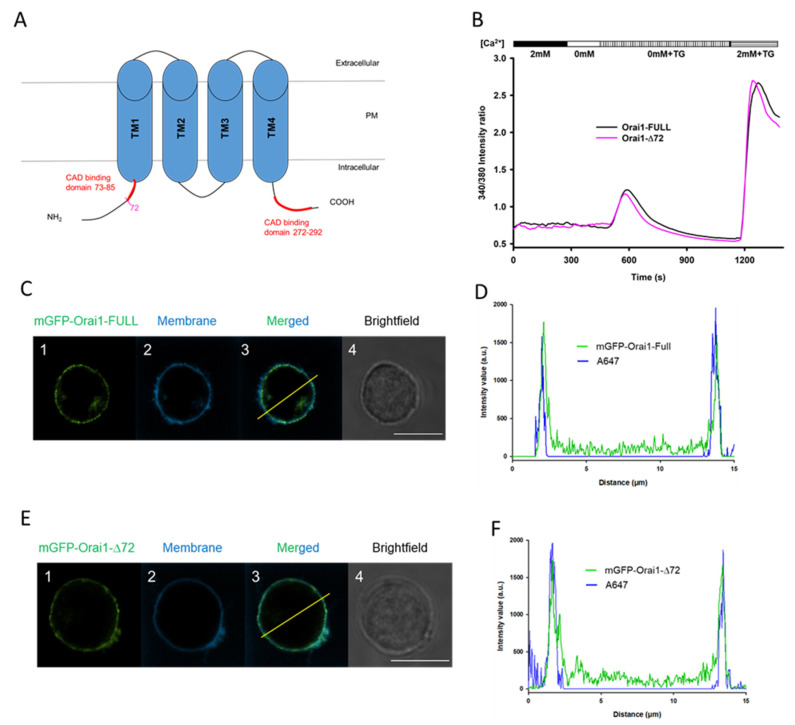
Removing the N terminal part of Orai1 (mGFP-Orai1-∆72) does not impact the channel expression and function. (**A**) Schematic illustration of the wild type full-length (Full) Orai1 channel in the plasma membrane with intracellular C- and N-terminal tails. CRAC activation domains (CAD) are labelled with red on the N terminal (73–85 aa) and on the C terminal (272–292 aa) and 72nd amino acid for the deletion mutant of Orai1 (mGFP-Orai1-∆72) is red. (**B**) Cytosolic Ca^2+^ measurements in Jurkat cells stably expressing the mGFP-Orai1-Full (black) and mGFP-Orai1-∆72 (pink) channels based on the 340 nm/380 nm intensity ratio. For the representative traces at least 63 cells were recorded per channel phenotype. (**C**,**E**) Representative confocal images of mGFP-Orai1-Full and mGFP-Orai1-∆72 channels stably expressed in Jurkat cells. (**C1**,**E1**): mGFP signal of mGFP-Orai1-Full and mGFP-Orai1-∆72 channels, respectively (green). (**C2**,**E2**): The Alexa Fluor™ 647 NHS Ester fluorescence of the plasma membrane (blue). (**C3**,**E3**): The merge of green and blue channels, note the overlap of signals. (**C4**,**E4**): Brightfield images of different Orai1 channels expressing cells. Scale bar is 10 µm for both image sets. The pixel-by-pixel intensity profile analysis of (**D**) mGFP-Orai1-Full and (**F**) mGFP-Orai1-∆72 channels via the line indicated in the merged image (**C3**,**E3**): green: mGFP-Orai1; blue: membrane, Alexa-647 NHS ester.

The C terminus of Orai1 subunit is essential for STIM1 coupling and SOCE activation, and N-terminal region contains a CAD binding domain that is necessary for the Ca^2+^ permeation [20,36,37,38,39]. Therefore, we created an N-terminal mutant named mGFP-Orai1-∆72 (mimicking Orai1β isoform and preserving N terminal binding of STIM1 partly), where the first 72 amino acids were removed to unveil the role of the intracellular amino-tail in the reorganization to the IS (Figure 4A).

Several studies reported that N-terminal truncations of Orai1 channel suppressed store-operated activation or completely abolished STIM1-dependent CRAC current activation [20,39]. Hence, the Ca^2+^ signaling of Jurkat cells stably expressing mGFP-Orai1-Full and mGFP-Orai1-∆72 channels was characterized as described above. The time-course of Ca^2+^ response by cells expressing mGFP-Orai1-Full and mGFPP-Orai1-Δ72 is shown in Figure 4B: truncation of the Orai1 (Orai1-Δ72) had no significant effect on the Ca^2+^ response of Jurkat cells, hence it did not influence significantly activation of the CRAC channel as described previously (Figure 4B) [25,40].

Next, we assessed the cellular localization of mGFP-Orai1-Δ72 construct in Jurkat cells: plasma membrane was stained with Alexa-647 succinimidyl ester and the line intensity profile was determined for the truncated channel expressing cells. Figure 4D,F clearly confirm the plasma membrane localization of Orai1 channel constructs: intensity profile analysis shows that intensity peaks of Alex647 staining (membrane labeling) and the mGFP-signal overlap just as for the wild-type Orai1 (see Figure 4C,E). Consequently, mGFP-Orai1-Δ72 channels are able to target the cell membrane.

**Figure 5:**
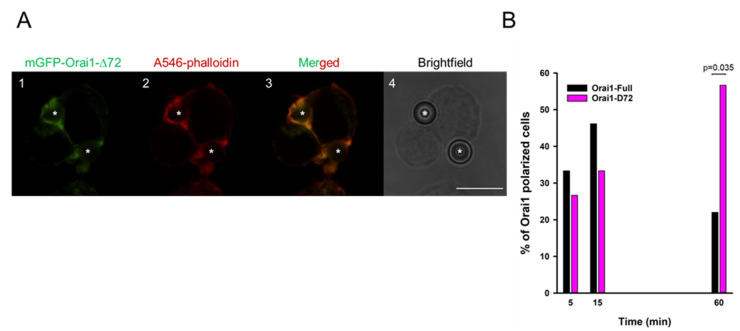
The mGFP-Orai1-∆72 are retained in the IS. Confocal snapshots of the immune synapse formed between Jurkat cells expressing mGFP-tagged Orai1-∆72 channel and CD3-CD28 antibody coated beads. (**A1**) mGFP-tagged Orai1-∆72 channel (green). (**A2**) Alexa-546 phalloidin (red) as an IS marker. (**A3**) Merged image of Orai1 (green) and Alexa-546 phalloidin (red) fluorescence signals. (**A4**) Brightfield image of the mGFP-Orai1-∆72 channel expressing cell show in Aa-Ac. The bead is indicated with an asterisk. Scale bar is 10 µm. (**B**) The percentage of cells showing the Orai1 polarization in mGFP-Orai1-Full (black, same data shown in Figure 1B) and in mGFP-Orai1-∆72 (pink) expressing cells at 5th, 15th and 60th minute. 30 cells were evaluated at each time point.

Furthermore we formed IS between Jurkat T cells stably expressing N-terminally truncated Orai1 channels and CD3-CD28 antibody coated beads (we included 5, 15, 60 min in the analysis). Representative confocal snapshots demonstrate that mGFP-Orai1-Δ72 could redistribute into the IS (Figure 5A, for mGFP-Orai1-Full expressing cells in IS see Figure 1A). Stunningly, the kinetics of Orai1 subunit IS-accumulation was different from the wild-type (Figure 1B and Figure 5B): the percent of truncated Orai1-expressing mGFP-polarized cells gradually elevated and reached a peak at 60th minute with 58% (*p* = 0.035 between mGFP-Orai1-Full and mGFP-Orai1-Δ72) similar to mGFP-Orai1-Full-STIM1-KD cells (Figure 3C). Also the AR value for mGFP-Orai1-Δ72 channels were significantly higher as compared to mGFP-Orai1-Full construct (*p* = 0.004, Figure S6).

### 2.4. SAP97 Could Control the Leaving of Orai1 from the Immunological Synapse

It was described before that the adapter protein SAP97 (synapse-associated protein 97) or hDlg1 redistribute to the IS and ca. 15 min after stimulation it is no longer enriched at the contact zone between the T cell and the CD3-CD28 antibody coated beads [41]. SAP97, a member of MAGUK family proteins (Membrane Associated Guanylate Kinases), plays role in targeting of membrane ion channels and cytosolic proteins [13,42,43,44]. Since wild-type mGFP-Orai1-Full channels have the maximum presence at 15 min in the synapse (and it is coupled to SAP97 via its N terminus, Figure S5), we propose that SAP97 might guide the removal of Orai1 from the synaptic region.

**Figure 6:**
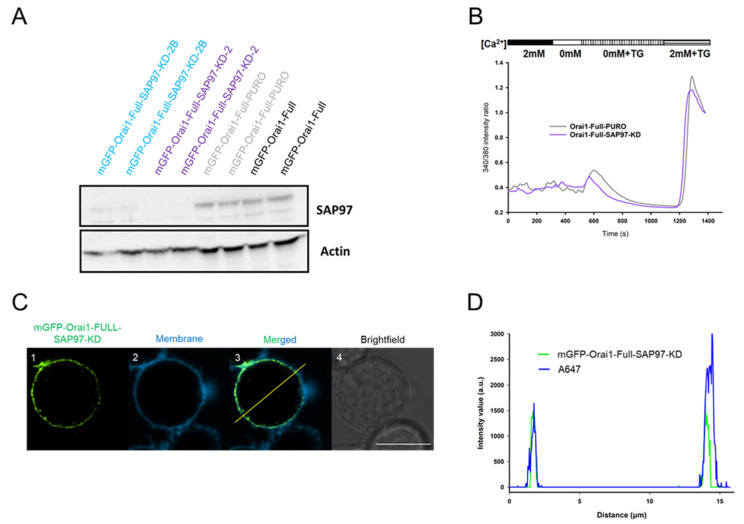
Knockdown of SAP97 in Jurkat cells preserve SOCE and Orai1 membrane targeting. (**A**) Western blot experiments of mGFP-Orai1-Full expressing Jurkat (black), mGFP-Orai1-Full-PURO (grey), mGFP-Orai1-Full-SAP97-KD2 (purple) (later we used this cell line and named it mGFP-Orai1-Full-SAP97-KD cell line) and mGFP-Orai1-Full-SAP97-KD2B cells (cyan) probed with a specific antibody against SAP97 (the expected size 130 kDa, each sample was loaded in duplicates). Actin was used as an expression control with an approximate size of 42kDa. (Knock-down efficiency was ca. 96%) Full-sized gel image is shown in Figure S3. (**B**) Cytosolic Ca^2+^ measurements using FURA-2 in mGFP-Orai1-Full-PURO (grey) as a transduction control and mGFP-Orai1-Full-SAP97-KD (purple) cells. The representative traces are the average of 18 cells (mGFP-Orai1-Full-PURO) and 21 cells (mGFP-Orai1-Full-SAP97-KD). (**C**) Confocal images of mGFP-Orai1-Full-SAP97-KD cells stably expressing mGFP-Orai1-Full channels: (**C1**) mGFP signal of Orai1-Full channel (green) in SAP97-KD cells. (**C2**) Alexa Fluor™ 647 NHS Ester fluorescence of the plasma membrane (blue). (**C3**) Fusion of green and blue channels, note that their overlap. (**C4**) Brightfield image of the mGFP-Orai1-Full-SAP97-KD cell in shown in Ca-Cc. Scale bar is 10 µm. (**D**) Pixel-by-pixel intensity profile analysis of mGFP-Orai1-Full channels in mGFP-Orai1-Full-SAP97-KD cell along the line indicated in the merged image (Cc): green: mGFP-Orai1-Full; blue: membrane, Alexa-647 NHS ester.

To unveil the role of the SAP97 in Orai1 trafficking to the IS we knocked down the SAP97 in Jurkat cells expressing mGFP-Orai1-Full channel (mGFP-Orai1-Full-SAP97-KD). As a control for transduction, the pLKO.1-puro plasmid was applied as previously (mGFP-Orai1-Full-PURO). The efficiency of SAP97 knock-down was tested using western blotting. In cell lines lacking the SAP97 (mGFP-Orai1-Full-SAP97-KD-2 or mGFP-Orai1-Full-SAP97-KD-2B) the intensity of SAP97 band was far lower as compared to transduction control (mGFP-Orai1-Full-PURO) or mGFP-Orai1-Full cells (Figure 6A). Based on these, we used the mGFP-Orai1-Full-SAP97-KD2 cell line for further experiments (referred as mGFP-Orai1-Full-SAP97-KD in the followings).

We checked the membrane expression of mGFP-Orai1-Full subunits by labeling the membrane with Alexa-647 succinimidyl ester. The intensity profile of Jurkat cells missing SAP97 and expressing the mGFP-Orai1-Full channels clearly demonstrates the membrane localization of the Orai1 subunit is not affected by the knockdown of SAP97 (Figure 6C,D). To test the functionality of Orai1 channels in mGFP-Orai1-Full-SAP97-KD cells, the cytosolic Ca^2+^ changes were measured using the ratiometric Ca^2+^ indicator FURA-2. The transduction of the mGFP-Orai1-Full cells with SAP97 shRNA construct had no significant effect on CRAC current in Jurkat cells compared to the mGFP-Orai1-Full-PURO cells (Figure 6B).

**Figure 7:**
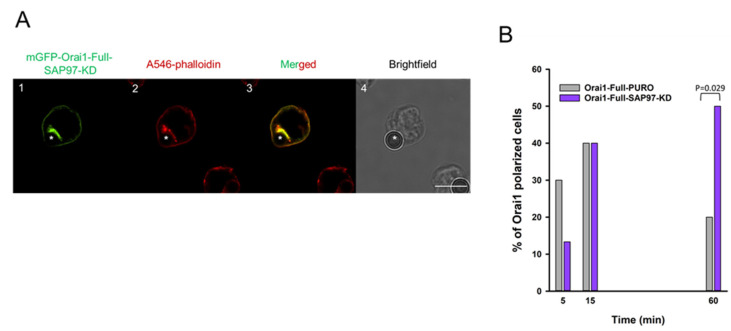
mGFP-Orai1-Full channels sustain relocation to the IS in the SAP97 knocked-down Jurkat cells (**A**) Confocal images of the immune synapse forming Jurkat cells expressing the Orai1-Full proteins in mGFP-Orai1-Full-SAP97-KD cells. (**A1**) mGFP signal of the Orai1-Full (green). (**A2**) Alexa-546 fluorescence of the phalloidin (red, IS indicator). (**A3**) Merged images of mGFP-Orai1-Full (green) and Alexa-546 phalloidin (red) fluorescence signals. (**A4**) Brightfield image of the mGFP-Orai1-Full channel expressing mGFP-Orai1-Full-SAP97-KD cell shown in Ca-Cc. Bead is indicated with an asterisk. Scale bar is 10 µm. (**B**) The percentage of cells with mGFP-Orai1-Full polarization at 5th, 15th, and 60th minute in mGFP-Orai1-Full-PURO cells (grey, same data as in Figure 3C) and in mGFP-Orai1-Full-SAP97-KD cells (purple). 30 cells were used for evaluation at each time point.

Thereafter, the kinetics of Orai1-accumulation was determined by creating IS between Jurkat cells expressing mGFP-Orai1-Full channels with reduced SAP97 expression, and CD3-CD28 antibody coated beads (Figure 7A, for mGFP-Orai1-Full-PURO control see Figure 3B). In SAP97 knocked-down cells the percent of Orai1-polarized cells at the 60th minute (Figure 7B, *p* = 0.029) was higher as compared to the control. This means that the redistribution kinetics is similar to the mGFP-Orai1-Full-STIM1-KD and mGFP-Orai1-Δ72 expressing cells (Figure 3C and Figure 5B) (AR value for Orai1 also showed significant difference between mGFP-Orai1-Full-SAP97-KD and mGFP-Orai1-Full-PURO control cells at 60th minute, *p* < 0.001, see Figure S6).

### 2.5. Sustained Orai1 IS-Residence Modifies Ca^2+^-Signaling

**Figure 8:**
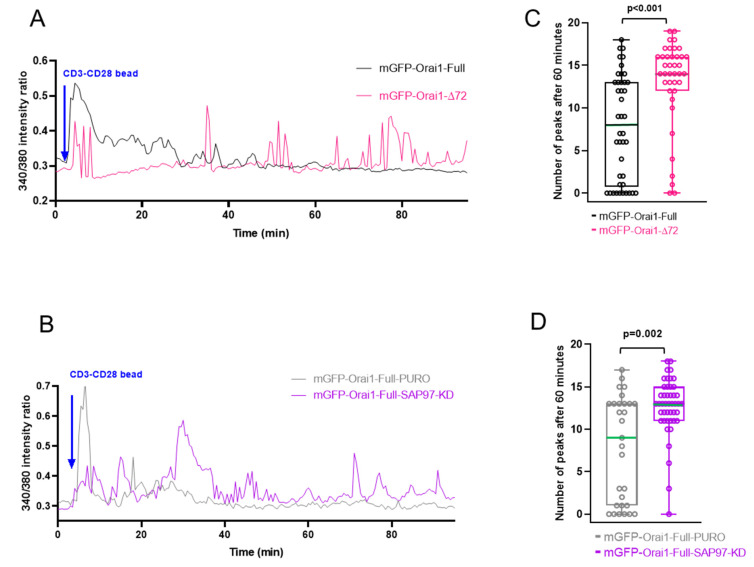
Sustained IS residency leads to calcium oscillation. Representative traces of cytosolic Ca^2+^ measurements using FURA-2 in Jurkat cells stably expressing (**A**) the Orai1 wild-type (mGFP-Orai1-Full), Orai1-N terminally truncated channels (mGFP-Orai1-∆72) (**B**) Orai1 wild-type missing SAP97 (mGFP-Orai1-Full-SAP97-KD), and Orai1 wild-type transfected with control shRNA (mGFP-Orai1-Full-PURO) upon forming immunological synapse with CD3-CD28 beads. Arrow indicates addition of CD3-CD28 beads. (**C**,**D**) Calcium response in different cell types between 60th and 90th minute after the IS was formed with beads (see materials and methods). For this experiment 42 cells of mGFP-Orai1-Full, 39 cells of mGFP-Orai1-∆72, 45 cells of mGFP-Orai1-Full-SAP97-KD and 29 cells of mGFP-Orai1-Full-PURO were used, from 3 independent experiments. Dots represent the number of calcium peaks between 60th and 90th minute for each cell. Green line indicates median.

To test the functional consequence of sustained Orai1 IS-residency we measured the Ca^2+^ responses in Jurkat cells forming immunological synapse with CD3-CD28 antibody coated beads. Our results showed increased number of calcium peaks in mGFP-Orai1-∆72 (compared to control mGFP-Orai1-Full, *p* = 0.016) and mGFP-Orai1-Full-SAP97-KD (compared to control mGFP-Orai1-Full-PURO, *p* < 0.001) during the first 60-min period of CD3-CD28 bead engagement. After 60 min the calcium oscillations were still present in N-terminally truncated Orai1 and in Orai1-Full expressing SAP97-KD cells unlike in control cells: the number of oscillatory peaks were significantly lower in control cells (mGFP-Orai1-∆72 vs. mGFP-Orai1-Full with *p* < 0.001; mGFP-Orai1-Full-SAP97-KD vs. mGFP-Orai1-Full-PURO with *p* = 0.002, between the 60th and the 90th minute) (Figure 8C,D).

Moreover, the time-course of Ca^2+^ response was altered: while control cells (mGFP-Orai1-Full, mGFP-Orai1-Full-PURO) usually had a main peak right after the bead conjugation, which subsided over time, in mGFP-Orai1-∆72 and mGFP-Orai1-Full-SAP97-KD cells several Ca^2+^ peaks was observed during first 60 min of IS and even after the 60th minute (Figure 8A,B).

## 3. Discussion

In this study, we assessed the accumulation kinetics of the mGFP-Orai1 channel into the IS and its molecular background. We demonstrated that the accumulation of Orai1 channels peaked 15 min after the IS formation, then the fraction of cells showing Orai1-polarization decreased up to the 60th minute. On the contrary, N-terminal truncated mGFP-Orai1-∆72 constructs in “native” Jurkat cells and mGFP-Orai1-Full subunits expressed in STIM1/SAP97 knockdown T cells had a high fraction of Orai1-polarized cells upon engagement to CD3-CD28 beads even at the 60th minute. Truncation of Orai1 N-terminal region (mGFP-Orai1-∆72) and the knockdown of SAP97 in mGFP-Orai1-Full-SAP97-KD cells had no significant effect on the Ca^2+^ current via CRAC channels. However, STIM1 knock-down abolished the Ca^2+^-response in Jurkat cells. Ca2+-signaling evoked by a CD3-CD28 bead in mGFP-Orai1-∆72 and mGFP-Orai1-Full-SAP97-KD cells were different as compared to corresponding control cells: the former showed persistent Ca^2+^ oscillations after 60th minute.

Several groups reported that various ion channels (Kv1.3, KCa3.1, Orai1 along with STIM1, P2X7, pannexin etc.) translocate to the IS between a T cell and an APC [13,14,15,45]. Upon T cell activation, the presence of CRAC channels in the contact zone can be necessary for providing the influx pathway for calcium and the potassium channels maintain the negative membrane potential and generate the driving force for calcium entry resulting in the proliferation of T cells [12,14,15,24]. The residence time of these different ion channels in the IS is essential for shaping the Ca^2+^ dependent activation. Nicolaue et al. showed that early leave of Kv1.3 channels could be a cause of altered Ca^2+^ signaling of SLE (systemic lupus erythematosus) T cells [30]. Here we showed for the first time, that Orai1 subunit of CRAC channels shows a biphasic accumulation. This kinetics might be essential to avoid sustained NFAT translocation to the nucleus and persistent activation, which can easily lead to hyperactivity of a T cell.

For monitoring Orai1 distribution and Ca^2+^ imaging in the IS we used CD3-CD28 antibody coated beads as an APC rather than cells, crosslinked antibodies or antibody coated coverslips generating a planar synapse. This method is commonly used and accepted for creating immune synapse [30,45,46]. Furthermore, antibody stimulation experiments by several groups revealed that Ca^2+^- response of T cells induced by antibody crosslinking or bead-conjugation is very different. Thus, we think that simply adding antibodies to cells or attaching cells to a planar surface is not sufficient: the presence of a CD3/CD28 bead attached to the cell is required for characteristic Ca^2+^ response of T cells. The IS formation can lead to reorganization of various cellular components, cell-polarization and change in cell shape shape (geometry), which is not likely to take place upon addition of antibodies or plating cells onto planar antibody coated surface.

To evaluate the kinetics of Orai1 IS-accumulation, we took snapshots at subsequent time points rather than performing live-cell imaging experiments. Based on work by Quintana et al., 2011 (live cell imaging vs. fixed cells showed similar Orai1 redistribution dynamics in the IS) [23], we suppose that with this method we could have well-controlled time intervals and selection of the appropriately oriented T cell-bead imaging axis at substantially better resolution than full frame imaging of the whole field of view. Statistically speaking the analysis of snapshots should result in the same distribution of Orai1 in the synapse as continuous imaging.

The method we applied to decide if a cell shows Orai1 polarization/accumulation on one hand was adapted from studies published by Nyakeriga et al., Nicolaou et al., Round et al. and Szilagyi et al. [13,30,47,48]. On the other hand, in a set of control experiments (CHO cells expressing mGFP tagged Kv1.3 proteins, no IS formation/standalone cells) we obtained that AR value (it defines how many times more the mGFP intensity in the area of IS is as compared to non-IS region) could be higher than 1.0 for some cells. To avoid the detection of false positive cases, we set the cutoff value to 1.5 for AR. With this threshold, we underestimate the number of Orai1-polarized cells, which could be regarded as Orai1 IS-accumulating cells, but exclude the false detection.

We determined the expression level of CD3 and CD28 in each cell line (native Jurkat E6-1, mGFP-Orai1-Full, mGFP-Orai1-∆72, mGFP-Orai1-Full-STIM1-KD, and mGFP-Orai1-Full-PURO) used in this study, and found that they have the same expression level (Figure S7). Based on this we are sure that changes we described here is not due to the “manipulation” of native Jurkat cells, instead the specific effect related to the change of Orai1 function and distribution.

During T cell activation, a specific antigen binding to the TCR/CD3 complex results in the IP3 binding to the IP3 receptors on the endoplasmic reticulum (ER) surface and releasing Ca^2+.^ Depletion of ER calcium stores is sensed by the ER-resident STIM1, which activates the pore-forming subunit, Orai1 evoking a calcium influx through CRAC channels. It was reported that STIM1 is accumulated with Orai1 in the IS [14,24]. To understand the function of STIM1 in the synapse and Orai1 trafficking, we knocked down STIM1 in Jurkat T cells expressing Orai1-Full channels (mGFP-Orai1-Full-STIM1-KD). As expected, the STIM1 knockdown completely abolished Ca^2+^-response of cells through CRAC channels. By monitoring the IS accumulation of Orai1 in Jurkat cells with low STIM1 expression, we could show that Orai1 is able to redistribute to the synapse even with extremely low STIM1 (knock down efficiency ca. 90%), as it was reported by Quintana et al., where overexpressed Orai1 could accumulate in the IS when exogenous STIM1 was not co-expressed [23]. The kinetics of Orai1 redistribution in mGFP-Orai1-Full-STIM1-KD cells was different from the mGFP-Orai1-Full-PURO cells: the percentage of Orai1 polarized T cells had the maximum at 60th minute after IS formation indicating the sustained rearrangement of Orai1 channels into the contact zone between the Jurkat cells and CD3-CD28 antibody coated beads. These findings, i.e., the Orai1 redistribution to the IS does not require the calcium flux through Orai1 for targeting into the IS, and Orai1 can relocate to the IS in the absence of STIM1 are consistent with results of Lioudyno et al.: non-functional CRAC channels do not prevent IS formation and Orai1, STIM1 and TCR can redistribute to the contact zone of the immunological synapse even without the Ca2+ influx through CRAC channels [14].

It was shown that Orai1 has two isoforms, the shorter (or beta) almost lacks the entire N terminal region before the ETON region required for Orai1 conductance and activation. An essential physiological consequence of the shorter isoform expression has been found so far is that beta isoform is unable to activate nuclear translocation of NFAT, however, these two isoforms have the same Ca^2+^-response upon thapsigargin stimulation (Kar et. al) [49]. Using the mGFP-Orai1-∆72 mutant, we demonstrated that removal of N-terminus has a dramatic effect on the IS-accumulation of Orai1, which probably has an influence on T cell activation. Based on this, we assume that beta isoform is probably able to tune T cell activation via affecting NFAT modulation.

Based on the similar accumulation kinetics of Orai1 subunits in mGFP-Orai1-Full-STIM1-KD and mGFP-Orai1-∆72 cells, we tested the hypothesis if SAP97 (or hDlg1), which redistributes to the IS then moves out 15 min after APC-engagement, could regulate the removal of Orai1 from the synaptic region. Upon knockdown of SAP97 we could observe (as for mGFP-Orai1-∆72 and mGFP-Orai1-Full in STIM1-KD Jurkat cells) a continuous relocation of mGFP-Orai1-Full proteins to the IS up to the 60th minute: this points out that SAP97 and STIM1 could be crucial in regulation of Orai1 residence in the synapse.

We mentioned above that fraction of cells showing Orai1 accumulation in the IS is higher at 60 min than at 15 min for mGFP-Orai1-∆72 and mGFP-Orai1-Full-SAP97-KD (Figure 5B and Figure 7B). We also found that time course of Ca^2+^-response in mGFP-Orai1-∆72, mGFP-Orai1-Full-SAP97-KD cells is distinct from that of the controls. Though, in the first 15 and 30 min there was no difference in the Ca^2+^ response of cell lines (Figure S4), during the first 60 min or between the 60th and the 90th minute we could show significant increase in spike frequency between control and “treated” (N-terminal truncated and SAP97-KD) groups. Since an IS between a T cell and an APC can last up to several hours (as described in [50]), the increased residency time of Orai1 (as seen without SAP97 or N terminus) could induce pathological pathways due to altered Ca^2+^-signaling.

Finally, we suppose SAP97, which can facilitate interactions between proteins and play role in endocytosis of ion channels [48], may be a part of a molecular complex: Orai1 is guided out of the IS via association with STIM1 and SAP97. This presumption is consistent with the similar accumulation kinetics of the N-terminal truncated Orai1 in Jurkat cells and mGFP-Orai1-Full channels in STIM1- and SAP97-knockdown cells and the alike Ca^2+^-spikes pattern of mGFP-Orai1-∆72 and mGFP-Orai1-Full-SAP97-KD cells.

## 4. Materials and Methods

### 4.1. Cell Culture

Jurkat cells were cultured in RPMI solution (Sigma-Aldrich Ltd., Budapest, Hungary) supplemented with 10% FBS, 2 mM L-glutamine, 1mM Na-pyruvate, and 200 units penicillin/streptomycin. HEK-293T cells were cultured in DMEM medium (Sigma-Aldrich Ltd., Budapest, Hungary), which also contained 10% FBS, 2 mM L-glutamine, 1 mM Na-pyruvate, and 200 units penicillin/streptomycin. Cells were maintained at 37 °C in a humidified atmosphere of 5% of CO_2_ and 95% air. Cells were passaged every 3 days.

### 4.2. Plasmids and Cloning

The wild type Orai1 channel (mGFP-Orai1-Full) obtained from Richard Lewis’ lab (Department of Molecular and Cellular Physiology, Stanford University, Stanford, CA, USA) and the N-terminally truncated Orai1 (mGFP-Orai1-∆72) channel, in which the first 72 amino acids were deleted, were tagged with an mGFP at the N-terminal region and subcloned into the retroviral pBMN–LacZ vector (obtained from Nolan’s Lab, Baxter Laboratory in Genetic Pharmacology, Department of Microbiology and Immunology, Stanford University, Stanford, CA, USA) using Hind III and Not I restriction sites (Thermo Fisher Scientific Inc., Budapest, Hungary). Briefly, the PCR-amplified constructs were run on 1% agarose gel, then cut and purified with phenol extraction method. Afterwards, both the insert and the plasmid were digested with Hind III and Not I enzymes and cleaned up using ethanol precipitation methods. For sticky-end ligation, T4 DNA ligase was used according to the standard protocol (Biocenter Ltd., Szeged, Hungary). All constructs were sequenced at the Clinical Genomics Center at University of Debrecen.

### 4.3. Membrane Targeting

Jurkat cells expressing the mGFP-Orai1-Full, mGFP-Orai1-∆72, and genetically modified cell lines (mGFP-Orai1-Full-STIM1-KD, mGFP-Orai1-Full-PURO and mGFP-Orai1-Full-SAP97-KD cells, see knockdown of STIM1 and SAP97) expressing the mGFP-Orai1-Full channel were put into glass bottom chamber slides and were labelled with Alexa Fluor™ 647 NHS (succinimidyl) ester in 1 × TBS (Tris-buffered saline; 25 mM Tris–HCl, 150 mM NaCl, pH = 7.5) for 20 min on ice. NIKON T*i*2 microscope was used to take confocal images of the cells. Based on the fluorescent intensity of the GFP and Alexa Fluor™ 647 NHS Ester, the overlapping part of the signals that demonstrates the membrane localization was determined by using the Intensity Plot Profile tool of the ImageJ software.

### 4.4. Western Blotting

Protein samples were separated by SDS–PAGE and transferred to PVDF membranes (Millipore, Billerica, MA, USA) after electrophoresis. The membranes were blocked with milk powder and immunoblotted with mouse anti-STIM1 (MA1-19451, Sigma-Aldrich, Budapest, Hungary) or mouse anti-SAP97 (BioLegend, MMS-5187) and rabbit anti-actin (PA1-46296, Thermo Fisher Scientific Inc., Budapest, Hungary) primary or and anti-mouse IgG HRP-linked or anti-rabbit IgG HRP-linked secondary antibodies (Cell Signaling Technology, Inc., Beverly, MA, USA), respectively. Blots were developed with ECL reagent (Thermo Fisher Scientific Inc., Budapest, Hungary). The blots were then visualized with the FluorChem Q MultiImage III Western blot imaging system (ProteinSimple, San Jose, CA, USA). In each experiment 1 × 10^6^ cells were harvested.

### 4.5. Viral Transduction

Transduction protocol for Jurkat cells was obtained from the Nolan’s Lab web page (http://www.stanford.edu/group/nolan/, assessed on 1 August 2010).

### 4.6. Knockdown of STIM1 and SAP97 with shRNA

STIM1 and SAP97 knockdown Jurkat cells were obtained using the retroviral transduction protocol adapted from the Nolan’s Lab using Mission shRNA by Sigma-Aldrich Ltd., Hungary. The shRNA sequence was the following for STIM1: CCGGTGGTGGTGTC TATCGTTATTGCTCGAGCAATAACGATAGACACCACCATTTTTG (mGFP-Orai1-Full-STIM1-KD), and for the SAP97: CCGGCGGGTCAATGACTGTATATTACTCGAGTAA TATACAGTCATTGACCCGTTTTT (mGFP-Orai1-Full-SAP97-KD). For transduction/knock-down control, we used the pLKO.1-puro plasmid vector (Sigma-Aldrich, Budapest, Hungary) which contains an shRNA insert that does not target any known genes from any species (mGFP-Orai1-Full-PURO). The knock-down efficiency was assessed using of ImageJ software densitometry procedure.

### 4.7. Immunological Synapse Formation and Immunofluorescence

IS was formed between Jurkat T cells stably expressing the Orai1 wild-type (mGFP-Orai1-Full), Orai1-N terminally truncated channels (mGFP-Orai1-∆72), Orai1 wild-type missing STIM1 (mGFP-Orai1-Full-STIM1-KD), Orai1 wild-type missing SAP97 (mGFP-Orai1-Full-SAP97-KD), Orai1 wild-type transfected with control shRNA (mGFP-Orai1-Full-PURO), and Dynabeads™ Human T activator CD3-CD28 beads (Thermo Fisher Scientific Inc., Budapest, Hungary). Cell-bead conjugates were formed by mixing Jurkat cells and CD3-CD28 beads in RPMI medium (same as for culturing), and co-centrifugating them at 200× *g* for 1 min at 37 °C. Then cell-bead mixtures were plated on poly-L-lysine coated coverslips and were incubated for 1, 5, 15, 30 or 60 min at 37 °C in a humidified atmosphere of 5% of CO_2_ and 95% air, and were placed on ice for fixing. The cells were washed once with 1 × TBS (TRIS-buffered saline; 25 mM Tris–HCl, 150 mM NaCl pH = 7.5) containing 1% BSA and fixed with acetone for 1 min on ice. Between each step the cells were rinsed three times with 1 × TBS containing 1% BSA. F-actin accumulation at the IS was detected with Alexa-546 phalloidin (Thermo Fisher Scientific Inc., Budapest, Hungary), which was an indicator of IS formation. Finally, coverslips were mounted onto slides with Fluoromount G (eBioScience, San Diego, CA, USA).

Experiments with mGFP-Orai1-Full-PURO, mGFP-Orai1-Full-SAP97-KD and mGFP-Orai1-Full-STIM1-KD cells were performed in parallel.

### 4.8. Confocal Microscopy and Image Analysis

NIKON T*i*2 microscope (Plan Apo 60× water objective with numerical aperture 1.27) was used to take confocal images of the cells (the thickness of the slices was 1 μm). For excitation of mGFP the line 488 nm of Ar-ion laser, for A546 the line of 561 nm of He–Ne laser was used, and emitted light was detected through 505–550-nm bandpass, 550–640 nm bandpass filters, respectively. To determine the residency time of Orai1 channels in the IS, confocal images of Jurkat T cells engaged to CD3–CD28 beads were recorded at 1, 5, 15, 30, and 60 min time-points. The criterion of the IS formation was that T cell showed F-actin (A546-phalloidin) polarization at the bead-cell contact. For the evaluation of confocal images, we used the ImageJ software (National Institutes of Health, Bethesda, MD, USA): same-sized squares were drawn as ROIs at the IS (bead-cell contact area, according to F-actin polarization using A546-phalloidin signal (6 squares)), outside the IS (including the membrane and intracellular region, 3 squares) and at a cell-free area for the background (3 squares), respectively. The accumulation ratio (AR) was defined by the following equation (see Figure S1):

$$\mathrm{AR}\;=\frac{\mathrm{Mean}\;\mathrm{intensity}\;\mathrm{of}\;\mathrm{Orai}1\;\mathrm{in}\;\mathrm{IS}\;-\;\mathrm{Mean}\;\mathrm{intensity}\;\mathrm{of}\;\mathrm{background}}{\mathrm{Mean}\;\mathrm{intensity}\;\mathrm{of}\;\mathrm{Orai}1\;\mathrm{outside}\;\mathrm{the}\;\mathrm{IS}\;-\;\mathrm{Mean}\;\mathrm{intensity}\;\mathrm{of}\;\mathrm{background}}$$

Cells were defined Orai1-polarized if AR was higher than 1.5 (for details see Figure S1).

### 4.9. Intracellular Ca^2+^ Measurements

First, the Jurkat cells were plated onto poly-L-lysine coated glass bottom petri dishes. Then the cells were loaded with 1 μM FURA-2 acetoxymethyl ester (Thermo Fisher Scientific Inc., Budapest, Hungary) dissolved in DMSO and incubated for 25 min at 37 °C in phenol-red free RPMI solution (Sigma-Aldrich Ltd., Budapest, Hungary), supplemented with 10% FBS, 2 mM L-glutamine, 1 mM Na-pyruvate, and 200 units penicillin/streptomycin. The cells were washed with 2 mM Ca^2+^ solution (143.3 mM NaCl, 4.7 mM KCl, 10 mM HEPES, 5.5 mM glucose, 2 mM CaCl_2_, 1 mM MgCl_2,_ pH 7.35) and were placed on a 37 °C stage of an inverted fluorescence microscope. The cells were perfused with 2 mM Ca^2+^ solution, 0 mM Ca^2+^ solution (143.3 mM NaCl, 4.7 mM KCl, 10 mM Hepes, 5.5 mM glucose, 1 mM MgCl_2_, 0.1 mM EGTA, pH 7.35) and then 1 μM Thapsigargin (TG) (Thermo Fisher Scientific Inc.) containing 0 mM Ca^2+^ solution was applied to deplete Ca^2+^ stores via passive Ca^2+^ release from the endoplasmic reticulum (ER). After store depletion, the addition of extracellular 2 mM Ca^2+^ containing 1 μM TG activated intracellular Ca^2+^ elevation through SOCE (store operated calcium entry). Experiments with FURA-2 were completed with an inverted NIKON ECLIPSE T*s*2R microscope combined with a VisiChrome High Speed Polychromator (Visitron Systems GmbH, Puchheim, Germany). FURA-2 dual excitation and emission were accomplished using 340- and 380-nm excitation filters and a 510-nm emission filter. Digital images (100 ms exposure) were recorded with a PCO Edge 4.2 sCMOS Camera at 10 s intervals. Imaging data acquisition and analyses were accomplished using VisiView^®^ Imaging software. Only GFP expressing cells were selected for the evaluation.

### 4.10. Intracellular Ca^2+^ Measurements in IS

Jurkat T cells stably expressing Orai1 wild-type (mGFP-Orai1-Full), Orai1-N terminally truncated channels (mGFP-Orai1-∆72), Orai1 wild-type missing SAP97 (mGFP-Orai1-Full-SAP97-KD), Orai1 wild-type transfected with control shRNA (mGFP-Orai1-Full-PURO) were plated onto poly-L-lysine coated glass bottom petri dishes and were loaded with 1 μM FURA-2 acetoxymethyl ester (Thermo Fisher Scientific Inc., Budapest, Hungary) dissolved in DMSO and incubated for 25 min at 37 °C in phenol-red free RPMI solution (Sigma-Aldrich Ltd., Budapest, Hungary) supplemented with 10% FBS, 2 mM L-glutamine, 1 mM Na-pyruvate, and 200 units penicillin/streptomycin. The cells were washed with phenol-red free RPMI solution and were placed on a 37 °C stage of an inverted fluorescence microscope and the Ca^2+^ signal was recorded as described above (except that images were recorded at every 30 s). CD3-CD28 beads (Dynabeads™ Human T activator, Thermo Fisher Scientific Inc.) was pipetted onto Jurkat cells (mimicking immunological synapse) and Ca^2+^ responses were measured until 90th minute after contacting with beads. For evaluation, the Ca^2+^ signal in each cell was background corrected and the number of oscillations (peaks) in the 90-min period was determined. Those points were regarded as oscillatory peak that were higher than the average baseline (before adding the beads to cells) + 2 × SD (of the baseline), and height of the points before and after the peak was lower than the peak. Cells were measured on 3 different days. Only GFP expressing, “bead-activated” cells were selected for evaluation.

### 4.11. Statistical Analysis

To compare the percent of polarized cells we applied Fisher’s exact test. To compare the AR values and number of Ca^2+^-peaks for each sample we used one-way ANOVA on ranks or rank sum test. The level of significance was set to 0.05. For each time point and each cell line we collected data on N ≥ 3 days, and number of cells per day was *n* ≥ 9.

### 4.12. Reagents

All reagents were purchased from Sigma-Aldrich (St. Louis, MO, USA) unless stated otherwise.

## 5. Conclusions

In summary, we could show that the Orai1 subunit of CRAC channels have a unique accumulation kinetics in the IS. The knockdown of SAP97 and STIM1, and deletion of the putative binding site for SAP97 on Orai1 N terminus demonstrated that SAP97 and STIM1 may be essential in the regulation of Orai1 IS-residence. Arrest of Orai1 in the IS modify the Ca^2+^ response of Jurkat T cells upon CD3-CD28 activation, which possibly contributes to T cell hyperactivity. We suppose that existence of two Orai1 isoforms could have role in regulation of T cells’ Ca^2+^-dependent activation, however, this should be elucidated. We believe that learning the molecular background of Orai1 rearrangement to the IS can facilitate the understanding the mechanism of autoimmune diseases.

## Data Availability

We did not analyze public datasets in this manuscript.

## Supplementary Materials

The following are available online at https://www.mdpi.com/article/10.3390/ijms222111514/s1, Figure S1: Evaluation of Orai1 accumulation in the IS. Figure S2: Knockdown of STIM1 in Jurkat cells expressing mGFP-Orai1-Full channel. Figure S3: Knockdown of the SAP97 in Jurkat cells expressing mGFP-Orai1-Full channel. Figure S4: Calcium response analysis in different cell types in IS with CD3-CD28 beads Figure S5: SAP97 can bind to Orai1 via its N-terminus. Figure S6: Distribution of the accumulation ratio (AR). Figure S7: CD3 and CD28 expression in different cell lines.
