# Supplementary material for: Immune Synapse Residency of Orai1 Alters Ca2+ Response of T Cells
Source: Int J Mol Sci. 2021 Oct 26;22(21):11514. doi: 10.3390/ijms222111514 (PMC8583858; doi:10.3390/ijms222111514)
Supplement: Supplementary file 1 [file ijms-22-11514-s001.zip › ijms-1377540-supplementary.pdf]

Supplementary Figures and Tables

Supplementary materials and methods:

GST assay: The pGEX-4T-2 vector fused with glutathione S-transferase (GST) or pGEX-4T-2 containing the N terminal part of Orai1 (1-72aa, GST-Orai1-1-72aa) were produced in *Escherichia coli* (BL21). The bacterial lysates were induced with 1mM isopropyl beta-D-thiogalactoside (IPTG) for 2 hours in room temperature. The bacterial cells were harvested by centrifugation and washed 3 times with 1xPBS. They were sonicated in lysis buffer containing 50mM Tris-HCl pH7.5, 0.1% Tween20, 0.2% 2-mercaptoethanol and Protease Inhibitor Cocktail Set III (Calbiochem, Darmstadt, Germany) and were loaded on GST SpinTrap columns (GE Healthcare) for 1hour at 4°C. The Jurkat cells (containing the SAP97 proteins, 2×10^6) were washed 3 times with 1×PBS and sonicated in lysis buffer and were added to the GST columns and incubated for overnight at 4°C. The affinity columns were washed and the GST fusion proteins (GST and GST-Orai1-1-72aa) with Jurkat cells (containing SAP97) were eluated with 10mM glutathione. The eluates were separated by SDS–PAGE and transferred to PVDF membranes after electrophoresis and protein expression was confirmed by Western blotting using a specific antibody against SAP97 (Biolegend, UK).

Flow cytometry: Analysis of cells were performed with Novocyte flow cytometer system (ACEA Biosciences, San Diego, CA, USA) and NovoExpress software (ACEA Biosciences, San Diego, CA, USA), where at least 200,000 cells per sample were used. Immunostaining of different Jurkat cell lines (live cells, on 4 C) was carried out using Alexa Fluor 647 conjugated anti-human CD3 (SouthernBiotech, Birmingham, USA) or CD28 antibody (Biolegend, USA). Based on the dot plots created in NovoExpress software using statistics, the geometric mean and CV (coefficient of variation = SD/mean*100%) values are indicated for CD3 or CD28 in different cell lines. Only GFP-positive, live cells (FSC-SSC dotplot) were included in the evaluation and unlabeled Jurkat cells were applied as control.

Figure S1: Evaluation of the Orai1 accumulation in the IS

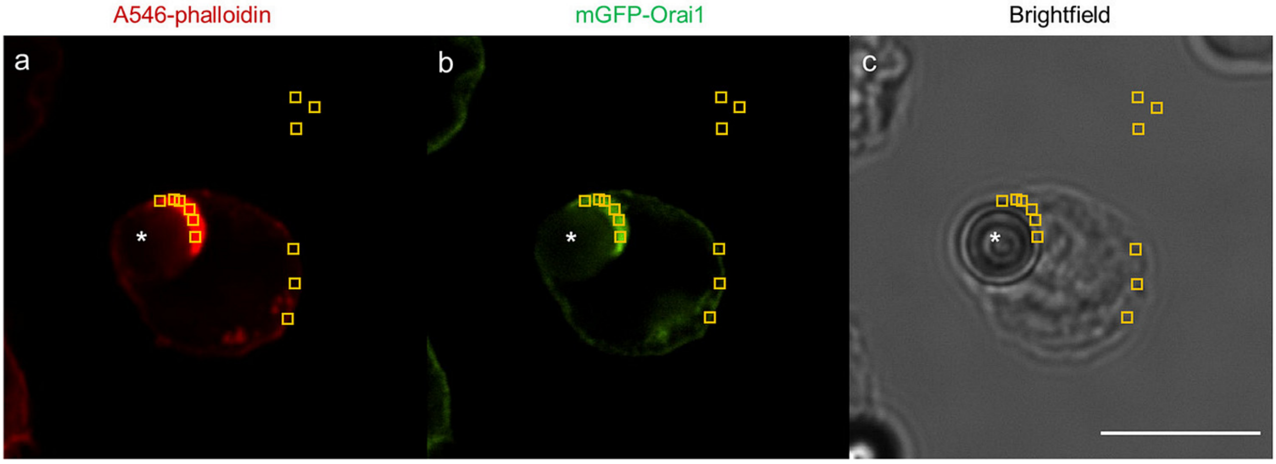

Figure S1: Evaluation of the Orai1 accumulation in the IS. For the evaluation of the residency kinetics in the IS, confocal images were taken of Jurkat cells expressing the different Orai1 channels and the **a)** A546-phalloidin (*red*) was used as an indicator to select the IS accumulation of the **b)** mGFP-tagged Orai1 channels (*green*). **c)** The brightfield images were used to determine the cell-bead position and assign the background area. Bead is indicated with an asterisk. Scale bar is 10 µm. Using ImageJ software same-sized squares (*yellow*) were drawn as ROIs in each picture in the same position at the IS, outside the IS on the cell and the background.

Figure S2: Knockdown of the STIM1 in Jurkat cells expressing mGFP-Orai1-Full channel

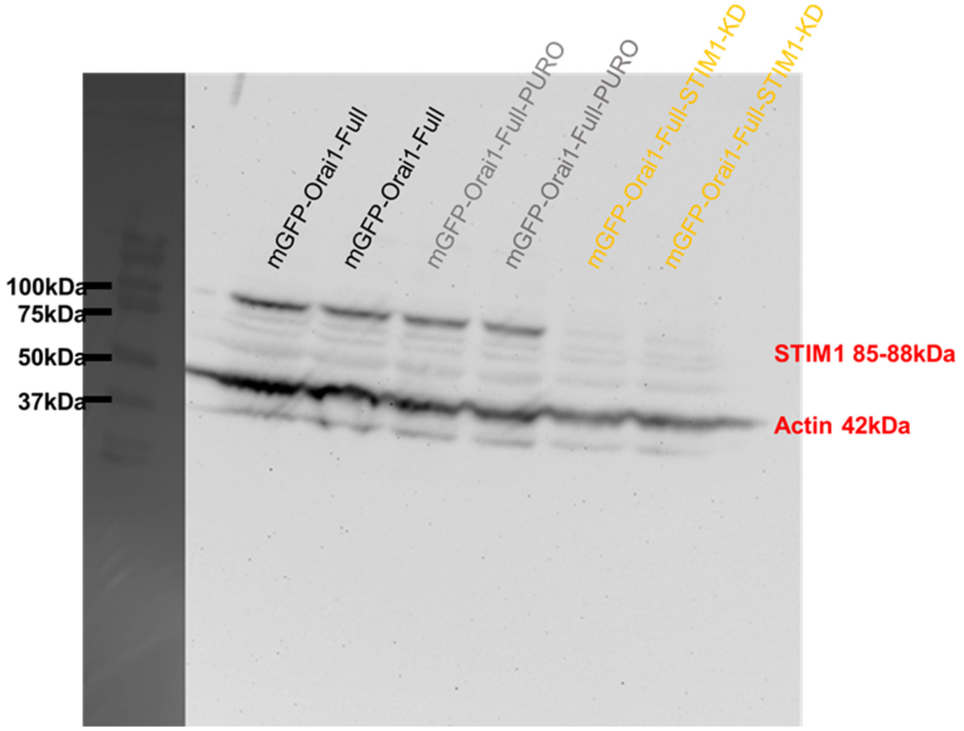

Figure S2: Knockdown of the STIM1 in Jurkat cells expressing mGFP-Orai1-Full channel. Western blot experiments of mGFP-Orai1-Full expressing Jurkat (*black*), mGFP-Orai1-Full-PURO (*green*) and mGFP-Orai1-Full-STIM1-KD (*blue)* cells probed with a specific antibody against STIM1 (the expected size 85-88 kDa). Actin antibody was used as an expression control with an approximate size of 42kDa. The marker was Precision Plus Protein Dual color standards marker (10 kDa – 250 kDa**).**

Figure S3: Knockdown of the SAP97 in Jurkat cells expressing mGFP-Orai1-Full channel

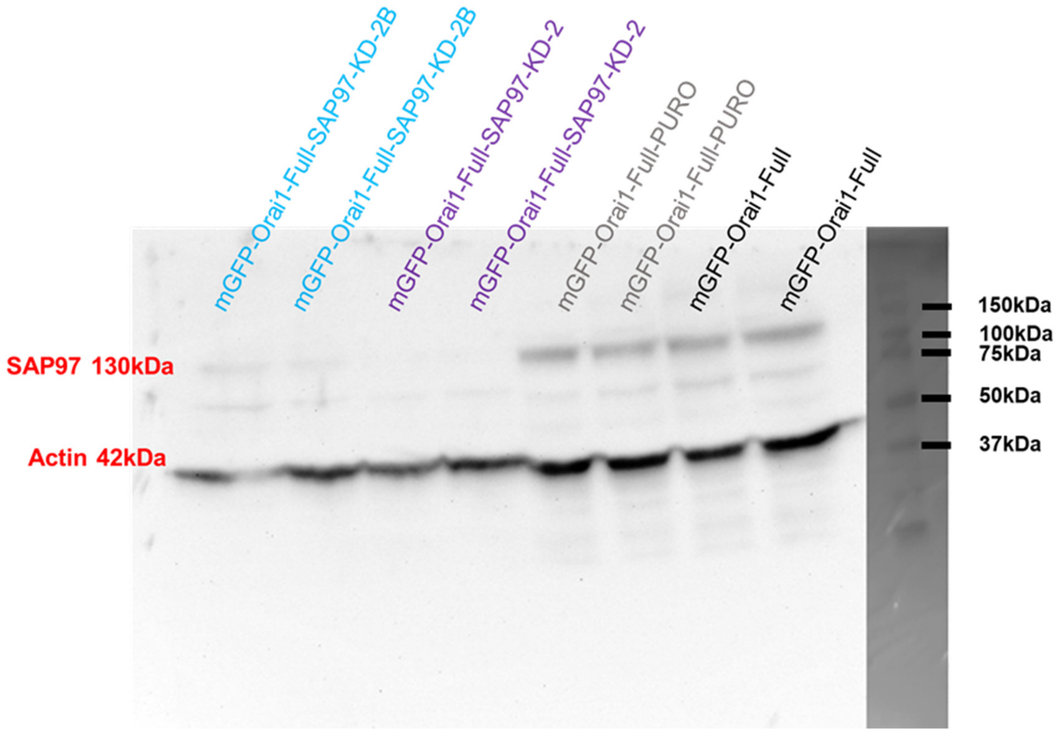

Figure S3: Knockdown of the SAP97 in Jurkat cells expressing mGFP-Orai1-Full channel. Western blot experiments of mGFP-Orai1-Full expressing Jurkat (*black*), mGFP-Orai1-Full-PURO (*grey*), mGFP-Orai1-Full-SAP97-KD2 (*purple)* (later we used these cells and named it mGFP-Orai1-Full-SAP97-KD cell line) and mGFP-Orai1-Full-SAP97-KD2B (cyan) cells probed with a specific antibody against SAP97 (the expected size 130 kDa). Actin was used as an expression control with an approximate size of 42kDa. The marker was Precision Plus Protein Dual color standards marker (10 kDa – 250 kDa**).**

Figure S4: Calcium response analysis in different cell types in IS with CD3-CD28 beads

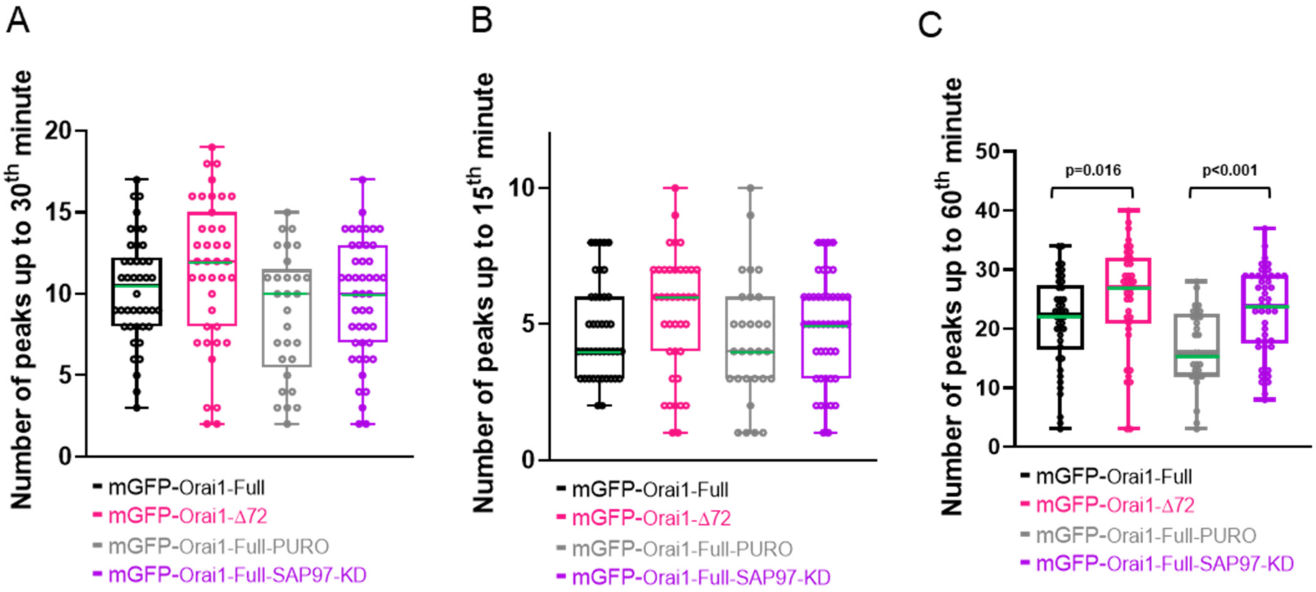

Figure S4: Calcium response analysis in different cell types in IS with CD3-CD28 beads. Calcium spikes measured in IS between Jurkat cells and CD3-CD28 beads (see materials and methods) up to **A)** 15 minutes, **B)** 30 minutes and **C)** 60 minutes. For these experiment 42 cells of mGFP-Orai1-Full, 39 cells of mGFP-Orai1-Δ72, 45 cells of mGFP-Orai1-Full-SAP97-KD and 29 cells of mGFP-Orai1-Full-PURO were used, from 3 independent experiments. Dots represent the number of calcium peaks for each cell. Green line indicates median.

Figure S5: SAP97 can bind to Orai1 via its N-terminus

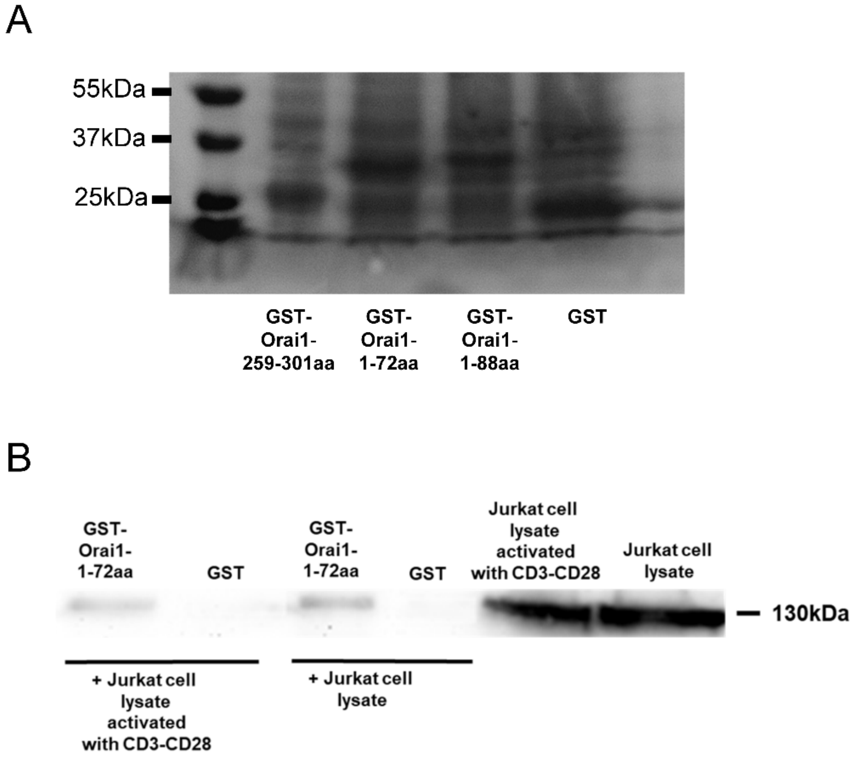

Figure S5: SAP97 can bind to Orai1 via its N-terminus. **A)** Coomassie-staining of bacterially expressed pGEX-4T-2 vector fused with glutathione S-transferase (GST-negative control) or pGEX-4T-2 containing the C-terminal part of Orai1 (GST-Orai1-259-301aa), a shorter N terminal part of Orai1 (GST-Orai1-1-72aa) and a longer a shorter N terminal part of Orai1 (GST-Orai1-1-88aa) proteins shows the efficiency of the purifications of bacterial extracts. The bacterial lysates were harvested by centrifugation, then washed, sonicated, and loaded on SDS-PAGE gel. **B)** Western blot analysis using SAP97 antibody of Jurkat cell lysates (positive control) or Jurkat cell lysates activated with CD3-CD28 and the eluted fractions of the pull-down experiments that were obtained by incubating total Jurkat cell lysates with the Sepharose-bead-immobilized fusion proteins (GST or GST-Orai1-1-72aa). The eluates were separated by SDS–PAGE and protein expression was confirmed by Western blotting. SAP97 was detectable in the eluate of GST-Orai1-1-72aa and in Jurkat cell lysates.

Figure S6: Distribution of the accumulation ratio (AR)

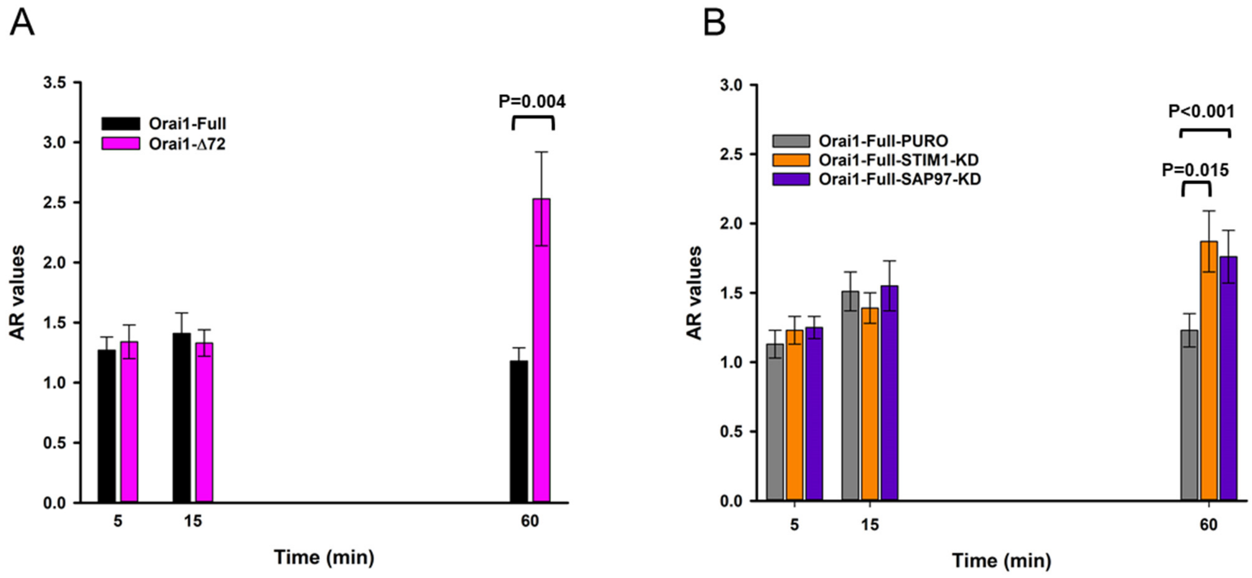

Figure S6: Distribution of the accumulation ratio (AR). AR value at indicated time points for **A)** Jurkat cells expressing mGFP-tagged Orai1-Full channels *(black)* and mGFP-Orai1-Δ72 *(pink)* and **B)** for mGFP-Orai1-Full-PURO *(grey)*, mmGFP-Orai1-Full-STIM1-KD *(orange)* and mGFP-Orai1-Full-SAP97-KD cells *(purple)* Data are presented as mean ± SEM (A: rank sum test, B,C: one-way ANOVA on ranks), statistical difference is indicated. These experiments were done in parallel.

Figure S7: CD3 and CD28 expression in different cell lines

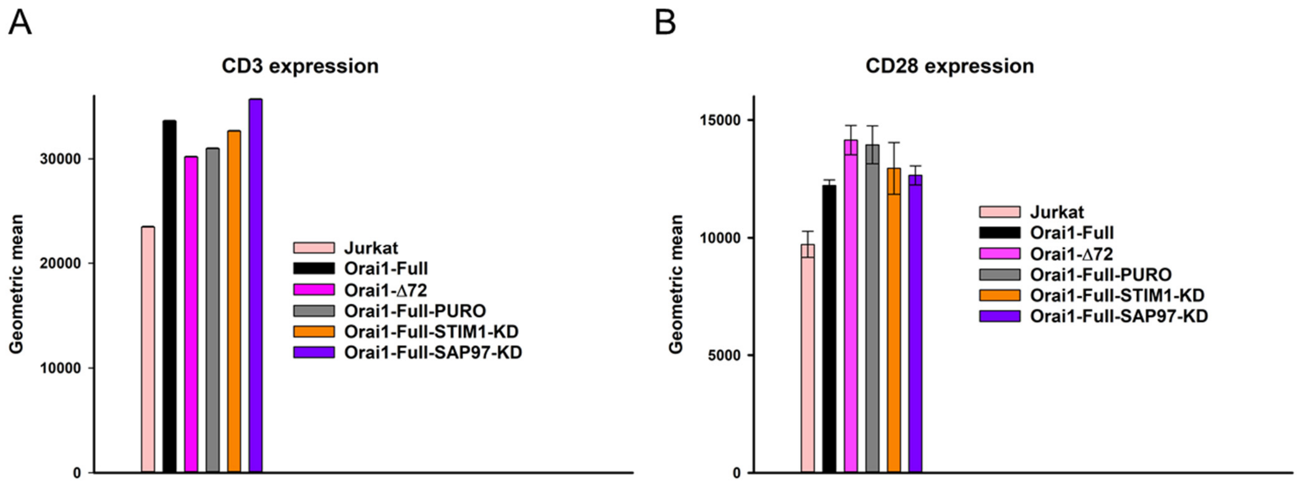

Figure S7: CD3 and CD28 expression in different cell lines. Immunostaining of different Jurkat cell lines was carried out using Alexa Fluor 647 conjugated **A)** anti-human CD3 or **B)** CD28 antibody. Analysis of cells were performed with Novocyte flow cytometer system (ACEA Biosciences, San Diego, CA, USA) and NovoExpress software (ACEA Biosciences, San Diego, CA, USA), where at least 200,000 cells per sample were used. Based on the dot plots created in Novoexpress software the geometric mean and CV values are indicated for CD3 or CD28 in different cell lines. (In panel **A** error bars are indicated but not visible due to the low values.) Only GFP positive, live cells were included in the evaluation.
